# EpCAM overexpression prolongs proliferative capacity of primary human breast epithelial cells and supports hyperplastic growth

**DOI:** 10.1186/1476-4598-12-56

**Published:** 2013-06-10

**Authors:** Agnieszka Martowicz, Johannes Rainer, Julien Lelong, Gilbert Spizzo, Guenther Gastl, Gerold Untergasser

**Affiliations:** 1 Laboratory of Experimental Oncology, Tyrolean Cancer Research Institute, Innsbruck, Austria; 2 Division of Molecular Pathophysiology, Biocenter, Innsbruck Medical University and Tyrolean Cancer Research Institute, Innsbruck, Austria; 3 Laboratory of Tumor Biology & Angiogenesis, Department of Internal Medicine V, Medical University of Innsbruck, Innsbruck, Austria

**Keywords:** EpCAM, p53, Primary mammary epithelial cells, CAM model, Transforming growth factor beta

## Abstract

**Introduction:**

The Epithelial Cell Adhesion Molecule (EpCAM) has been shown to be strongly expressed in human breast cancer and cancer stem cells and its overexpression has been supposed to support tumor progression and metastasis. However, effects of EpCAM overexpression on normal breast epithelial cells have never been studied before. Therefore, we analyzed effects of transient adenoviral overexpression of EpCAM on proliferation, migration and differentiation of primary human mammary epithelial cells (HMECs).

**Methods:**

HMECs were transfected by an adenoviral system for transient overexpression of EpCAM. Thereafter, changes in cell proliferation and migration were studied using a real time measurement system. Target gene expression was evaluated by transcriptome analysis in proliferating and polarized HMEC cultures. A Chicken Chorioallantoic Membrane (CAM) xenograft model was used to study effects on *in vivo* growth of HMECs.

**Results:**

EpCAM overexpression in HMECs did not significantly alter gene expression profile of proliferating or growth arrested cells. Proliferating HMECs displayed predominantly glycosylated EpCAM isoforms and were inhibited in cell proliferation and migration by upregulation of p27^KIP1^ and p53. HMECs with overexpression of EpCAM showed a down regulation of E-cadherin. Moreover, cells were more resistant to TGF-β1 induced growth arrest and maintained longer capacities to proliferate *in vitro*. EpCAM overexpressing HMECs xenografts in chicken embryos showed hyperplastic growth, lack of lumen formation and increased infiltrates of the chicken leukocytes.

**Conclusions:**

EpCAM revealed oncogenic features in normal human breast cells by inducing resistance to TGF-β1-mediated growth arrest and supporting a cell phenotype with longer proliferative capacities *in vitro*. EpCAM overexpression resulted in hyperplastic growth *in vivo*. Thus, we suggest that EpCAM acts as a prosurvival factor counteracting terminal differentiation processes in normal mammary glands.

## Introduction

EpCAM (also known as 17-1A, GA733-2, KSA, ESA, and EGP-40) is a homophilic, calcium-independent cell adhesion molecule of 39–42 kDa [1,2] expressed on most normal and cancerous epithelial tissues, cancer stem cells, embryonic stem cells and germ cells [3-5]. EpCAM is a type I transmembrane glycoprotein encoded by the *TACSTD1* gene. The EpCAM protein contains an extracellular domain (EpEX) with a nidogen-like domain as well as thyroglobulin- and epidermal growth factor-like repeats, a single transmembrane region, and a short intracellular domain (EpICD) consisting of 26 amino acids. EpCAM has been shown to be expressed on normal epithelial cells *in situ* at intercellular basolateral interfaces [1]. In regard to its function, it has been shown in the developing zebrafish, that EpCAM-lacking mutants display defects both in epithelial morphogenesis and epithelial integrity [1,6]. Moreover, mutants show abnormal skin development with higher infection susceptibility and enhanced skin inflammation [1,6]. In regard to mammals, EpCAM^-/-^ mice die in uterus at embryonic day 12, are developmentally delayed and display prominent placental abnormalities [7].

In tumor development and progression EpCAM has a controversial biological role [5]. As an adhesion molecule, EpCAM mediates homophilic cell-cell adhesion interactions thereby preventing metastasis [1,2]. In colorectal cancer EpCAM appears to act as molecule with protective function, since EpCAM deletions result in a higher risk to develop cancer [8] and overexpression of EpCAM in colorectal cancer cells has been shown to inhibit metastasis and invasion of tumor xenografts in mice [9].

On the other hand, it is known that EpCAM can abrogate E-cadherin mediated cell-cell adhesion thereby promoting metastasis [10]. Furthermore, it has been shown that EpCAM overexpression in cancer cells can support proliferation by enhancing Wnt signaling [11]. In breast carcinoma patients, high EpCAM expression was observed in less differentiated tumors [12] and was associated with larger tumors, nodal metastasis and worse survival of patients [13]. Moreover, high EpCAM expression correlated with poor prognosis in both node positive and node negative disease [14]. Due to its high expression in breast cancer tissue, EpCAM has emerged as an attractive target for treatment of breast cancer patients and recent studies with the humanized EpCAM antibody Adecatumumab showed already promising results in patients with EpCAM overexpression [15]. Moreover, the approval by the European Union in 2009 of the EpCAM-specific antibody Catumaxomab, adds a therapeutic option also in breast cancer patients with peritoneal carcinomatosis and malignant ascites [16].

Although it has been shown that EpCAM is expressed in normal epithelial cells [17] the role in normal breast tissue homeostasis is still unclear. In this study we analyzed effects of adenoviral overexpression of EpCAM on growth, migration and differentiation of normal breast epithelial cells. Moreover, we screened for genes altered by overexpression of EpCAM in normal epithelial cells of the breast and analyzed *in vivo* growth in a chicken xenograft model.

## Material and methods

### Tissue samples

A Human Breast Cancer Tissue Array, with matched metastatic carcinoma tissue (BR10010-2-BX), including TNM and pathology grade (50 cases, 100 cores) was purchased from Biocat and was composed of primary breast carcinoma (n = 50) with corresponding lymph node metastasis (n = 50). Samples from normal breast tissue (n = 5) were obtained in form of paraffin-embedded tissue block slides with normal breast tissue (Breast T2234086-BC). Detailed information about all tumor samples can be found on the supplier’s web site (http://www.biocat.com)

### Primary cell cultures (HMECs)

Human Mammary Epithelial Cells (HMECs, n = 4) were purchased from Promocell. HMECs were cultivated in Mammary Epithelial Cell Growth Medium with recommended supplements (Promocell, 0.004 mL/mL Bovine Pituitary Extract, 10 ng/mL Epidermal Growth Factor, 5 μg/mL Insulin and 0.5 μg/mL Hydrocortisone) on collagen-type-I (Sigma Biochemicals) coated ventilated plastic flasks. Cells were passaged by collagenase-type-I treatment (1 mg/mL, Sigma Biochemicals) and a cell detach kit (Promocell) consisting of 30 mM Hepes, 0.04%/0.03%Trypsin/EDTA Solution and Trypsin Neutralizing Solution (TNS). For TGF-β1 induced differention experiments cells were stimulated for 72 h with 1 ng/mL TGF-β1 recombinant human TGF-β1 R&D Systems in growth factor reduced medium. Cell numbers were determined 3 and 6 days after transfection and TGF-β1 stimulation by trypan-blue staining (Invitrogen) in the Buerker Tuerk counting chamber.

### MCF-10A cell line

Immortalized non-tumorigenic human mammary epithelial cells (MCF-10A) were obtained from the ATCC and cultivated in Dulbecco’s modified Eagle’s medium F12 (DMEM/F12) supplemented with 5% horse serum (both Invitrogen), 1% penicillin/streptomycin (PAA Laboratories GmbH), 0.5 μg/mL hydrocortisone, 10 μg/mL insulin and 20 ng/mL recombinant human EGF (all from Sigma Biochemicals).

MCF-10A^ns/ctrl^ and MCF-10A^E#2^ (control and EpCAM knockdown by shRNA) cell lines were generated by transfection with pGIPZ-shRNA-mir lentivirus as described elsewhere [18] and selected with 3 μg/mL puromycin (Invitrogen) for 5 days in standard culture medium.

### Generation of polarized cultures of HMECs

HMECs of 3 independent donors were seeded on a transwell 0.4 μm polyester membrane (Costar) coated with growth factor reduced matrigel (BD Biosciences, 50 μg/cm^2^). Cell culture medium was exchanged daily. Until day 4–5 cells formed confluent monolayer and until day 12 they polarized. The polarization status of the culture was confirmed by transepithelial resistance measurement using the STX2 electrode and the EVOM epithelial voltohmmeter (World Precision Instruments, WPI). For transepithelial resistance calculations we used the following formule: (measured value – blank value) × 18.1.

### Immunohistochemistry

Tissue sections were deparaffinized and hydrated in xylene and graded alcohol series. Antigen retrieval was performed in water bath (95°C) for 20 minutes with a target retrieval solution (Dako Cytomation) and endogenous peroxidase activity was blocked with 3% H_2_O_2_/methanol. Sections were incubated in blocking solution containing 10% bovine calf serum (Dako Cytomation) for 45 min and then stained for one hour with primary antibody (mouse anti-human EpCAM, ESA, clone VU-1D9, Novocastra, 1 μg/mL). Moreover, serial sections were incubated with a monoclonal mouse anti-human cytokeratin high molecular weight (clone 34ßE12; 1:100, Dako Cytomation), anti human cytokeratin 18 (clone DC-10; 1:50, Dako Cytomation), alpha smooth muscle cell actin (clone 1A4; 1:100, Sigma Biochemicals) and anti human p63 (clone 4A4; 1:50, Dako Cytomation). Primary antiserum was detected after incubation with a biotinylated secondary antibody (biotinylated rabbit anti-mouse IgG, Vector Laboratories Inc.) using the Vectastain Elite ABC Kit (Vector Laboratories Inc.) and the FAST DAB Tablet Set (Sigma Biochemicals). Sections were counterstained with Meyer’s hematoxylin and mounted with Pertex (Medite).

### Immunofluorescence

Cells were seeded on a Matrigel coated eight-well culture slides (Falcon BD Labware). Polarized 3D cultures cells were fixed, permeabilized and stained directly on Matrigel coated transwells. After being fixed in 4% paraformaldehyde and permeabilized with 0.2% Triton-X-100 cells were blocked with PBS containing 3% BSA for 45 min at room temperature (RT). All antibodies of the immunohistochemistry section and additional antibodies – anti human ZO-1 (clone 1/ZO-1, BD Biosciences Pharmingen), anti human E-cadherin (clone 32A8, Cell Signaling Technology), anti-β-catenin (clone 14/Beta-Catenin, BD Biosciences Pharmingen) were applied in a 1:100 dilution at RT for two hours. After washing in PBS cells were incubated with secondary fluorochrome-labeled antibodies (FITC-labeled/PE-labeled rabbit anti mouse, Dako Cytomation) and nuclei were counterstained with TO-PRO-3 Iodide or DAPI (both Molecular Probes). Cells were embedded in fluorescent mounting medium (Dako Cytomation) and viewed by a Fluorescence Microscope (Zeiss Axiovert 200, Carl Zeiss, Axiovision Software).

### Western blot analysis

Cells were harvested and lysed in a RIPA buffer (Sigma Biochemicals) containing protease inhibitors (Complete Mini EDTA-free; Roche Applied Science). 20 μg total protein was denaturated, separated by a 4-20% SDS-PAGE (Criterion TGX, Bio-Rad) and transferred to Immuno-Blot™ polyvinylidene difluoride (PVDF) membrane (Bio-Rad). After blocking the membrane in 5% non-fat milk powder dissolved in phosphate-buffered saline (PBS), membranes were incubated in 1% non-fat milk powder at 4°C overnight with primary mouse antibodies. Afterwards, membranes were incubated with a HRP-conjugated goat anti-mouse IgG (Dako Cytomation) diluted 1:1000. After washing, a chemoluminescent substrate (LumiGLO Reagent and Peroxide, Cell Signaling Technology) was added to the membrane, which was then exposed in the Chemidoc XRS station (Biorad Laboratories). Antibodies used for Western analysis were C-10 (mouse monoclonal against human EpCAM, Santa Cruz Biotechnology), alpha tubulin (clone B5-1-2; Sigma Biochemicals), E-cadherin (clone 32A8, Cell Signaling Technology), Vimentin (clone v9, Dako Cytomation), Cytokeratin 18 (clone DC 10, Dako), Cytokeratin, High Molecular Weight (clone 34ßE12, Dako Cytomation), p27^Kip1^ (clone G173-524, BD Pharmingen) and p53 (clone PAb1801, Calbiochem).

### PNGaseF treatment

Enzymatic deglycosylation of total protein (20 μg) was performed with PNGaseF enzyme (New England Biolabs) according to manufacturer’s protocol. Thereafter, protein extracts were analyzed by Western Blot.

### Adenoviral overexpression of EpCAM

Replication-defective adenoviruses were generated with the Ad-Easy Adenoviral vector system (Stratagene) according to the manufacturer’s instructions and as described elsewhere [19]. In brief, the EpCAM cDNA (NM_002354, Openbiosystems) was subcloned into the pShuttle CMV GFP vector and sequenced. Recombinant adenoviral DNA was generated in *BJ5183 bacteria cells* using a double-recombination event between cotransfected adenoviral backbone plasmid vector, pAdEasy-1, and a shuttle vector carrying the gene of interest. For generation of replication-defective adenovirus recombinant DNA was transfected into HEK293 cells using Lipofectamin 2000 (Invitrogen). All viral titers were determined by qPCR for the gene coding for the encapsulation signal (for:5-cgacggatgtggcaaaagt, rev: 5-cctaaaaccgcgcgaaaa) and the respective viral plasmid DNA standards. HMECs were transfected with a multiplicity of infection (MOI) of 100 viruses/cell and tested for gene and protein expression 24 to 118 hours after transfection. All cell proliferation, migration and *in vivo* assays were performed at least 24 hours after adenoviral transfection to allow efficient EpCAM overexpression.

### Flow cytometry

For FACS analysis of membranous EpCAM staining, cells were washed in PBS, resuspended and incubated with the first anti-EpCAM antibody (sc-25308, Santa Cruz Biotechnology, 1 μg/mL) for 30 min and then with the second, PE-labeled antibody (PE-labeled rabbit anti mouse, Dako Cytomation). Thereafter, cells were washed with PBS, resuspended and stainings were evaluated by a FACSCalibur (Becton-Dickinson, Heidelberg, Germany).

HMEC cell death was evaluated by human APC-labelled Annexin V (Alexis Biochemicals) and propidium iodide (PI, Sigma Biochemicals) stainings. Cells were adenovirally transfected (MOI = 100) and incubated for 24 hours. Thereafter, cells were resuspended in 200 μl Annexin V Binding Buffer (abcam) with 5 μL of Annexin V and 2 μL of PI (20 μg/mL), incubated for 15 minutes on ice, washed and resuspended in PBS/5% FCS prior the analysis. Cells were examined in the FACSCalibur (Becton-Dickinson, Heidelberg, Germany).

### Microarray data set generation and analysis

Gene expression profiling analysis was performed at the Expression Profiling Unit of the Medical University Innsbruck. RNA quantity was determined by optical density measurements and RNA integrity using the 2100 Bioanalyzer (Agilent Technologies, Palo Alto, CA). Fifty ng high quality RNA were processed using the WT Expression Kit (Ambion) and the WT Terminal Labeling Kit (Affymetrix). The resulting biotinylated targets were hybridized to Affymetrix Human Gene ST 1.0 v microarrays. Microarrays were washed and stained in an Affymetrix fluidic station 450, fluorescence signals were recorded by an Affymetrix scanner 3000 and image analysis was performed with the GCOS software (Affymetrix). Raw and preprocessed microarray data have been deposited at the Gene Expression Omnibus accession number GSE37172 and GSE39071. http://www.ncbi.nlm.nih.gov/geo/query/acc.cgi?token=xhyxvqggwgewqts&acc=GSE37172http://www.ncbi.nlm.nih.gov/geo/query/acc.cgi?token=hxspfguwciquuhg&acc=GSE39071.

### Quantitative RT-PCR analysis

Total RNA was isolated from HMECs using the TriReagent (Sigma Biochemicals), according to manufacturer’s instructions. For microarrays, RNA was purified by cell lysis and nucleic acid extraction using the RNeasy Kit (Qiagen). Thereafter, viral and genomic DNA in the RNA samples was digested with the RQ1 DNAse (Promega). The cDNA was amplified from 1 μg total RNA by the use of the SuperScript II Reverse Transcriptase Kit (Invitrogen Life Technologies). For validation, real time RT-PCR was performed using a SensiMix SYBR No-ROX Kit (Bioline) and a Rotor-Gene 6000 detection system (Corbett Research). Primers were designed to amplify specific GAPDH (for: 5-CTGACCTGCCGTCTAGAAAA; rev: 5-GAGCTTGACAAA GTGGTCGT), TATA Box Binding Protein (for: 5-GGAGCCAAGAGTGAAGAACA; rev: 5-AGCACAAGGCCTTCTAACCT) and EpCAM (for: 5-GCTGGTGTGTGAACACTGCT; rev: 5-ACGCGTTGTGATCTCCTTCT).

### Real time cell proliferation and migration assay (xCelligence system)

Real time cell proliferation and migration experiments were performed using the RTCA DP instrument (Roche Diagnostics GmbH), which was placed in a humidified incubator maintained at a 5% CO_2_ at 37°C. For proliferation assay cells were seeded in complete medium in 16-well plates (E-plate 16, Roche Diagnostics GmbH) at density of 5,000 cells/well. The plate containing gold microelectrodes on its bottom was monitored every 10 minutes for 4 hours (adhesion process), then once every 30 min, until the end of experiment, which was in total 72 hours (cell proliferation). Cell migration was performed using special 16-well plates with 8 μm pores (CIM-plate 16, Roche Diagnostics GmbH). These plates, resembling conventional transwells, have microelectrodes placed on the underside of the membrane. Cells were seeded into the upper chamber at a density of 20,000 cells/well in a serum free medium and the lower chamber was filled with complete medium. The plate was monitored every 15 minutes for 12 hours. Data analysis was performed using RTCA software 1.2 supplied with the instrument.

### Senescence associated-beta galactosidase (*SA*-*β*-*gal*) activity assay

Cells were fixed (2% formaldehyde, 0.2% glutaraldehyde in PBS) for 5 min at room temperature and rinsed several times in PBS. To measure *SA*-*β*-*gal* activity, cells were incubated in a staining-solution (4.2 mM citric acid, 12.5 mM sodium-phosphate, 158 mM sodium chloride, 0.21 mM magnesium chloride, 2.21 mg/mL potassium ferrocyanid, 1.68 mg/mL potassium ferricyanid, 1 mg/ml X-Gal, pH 6.0) for 24 h at 37°C. Cells were washed and embedded in PBS, viewed in an inverted transmission-microscope and photographed (Zeiss Axiovert 200, Axiovision software).

### Chicken chorioallantoic membrane (CAM) xenograft model

On embryo development day 0 fertilized chicken eggs (Gallus domesticus, Charles River) were placed in a 75-80% humidified 37°C incubator (Grumbach) to allow normal embryo development. On day 3 eggs were opened, egg shells removed and embryos were placed in a sterile Petri dish in an egg incubator to induce CAM development. On day 8, when chorioallantoic membrane (CAM) and its vasculature were well developed, all experiments were performed. HMECs were transfected one day before the experiment either by EpCAM adenoviruses or GFP control adenoviruses (both MOI = 100). 3.0 × 10^5^ cells were resuspended in a 30 μL drop of ice-cold growth-factor reduced Matrigel (Becton Dickinson) containing TGF-β1 in a concentration of 1.7 ng/mL and the mixture solidified for 30 min at 37°C. Subsequently, 4 onplants per chicken were grafted on the CAM. Growth of HMECs onplants was inspected on a daily basis using a stereo fluorescence microscope (Olympus SZW 10). On day 6 post-grafting chicken embryos were sacrificed with hypothermia, xenografts cut out and stored either in 4% paraformaldehyde for immunohistochemical studies or in TRI-reagent (Sigma Biochemicals) for RNA isolation.

### Statistical analyses

Statistical analyses were performed with the GraphPad Prism™ 5.0 (GraphPad Software, Inc.) software for Windows. All tests of statistical significance were two-sided. Student’s *T* test, two-way ANOVA and Mann–Whitney U Tests were used to study differences between two groups. Statistical analyses of quantitative PCR data were performed according to the delta Ct method described by Pfaffl *et al.*[20] and p values were calculated with the Student’s *T* Test. Data analysis of microarrays was performed in R (http://www.r-project.org) using packages from the Bioconductor project. The custom Ensembl transcript based CDF package (v13) from the brainarray group was used for probe set definitions. GeneChip raw expression values were preprocessed using the RMA method. After preprocessing a representative transcript probe, the set was selected for each gene as described previously [21]. In brief, a combination of average and variation of expression of a probe set across all samples was used to select the most informative transcript probe set for a gene. The moderated *t*-test was employed to assess significance of differential expression of a probe set between EpCAM overexpressing and control samples. The resulting raw p-values were adjusted for multiple hypotheses testing with Benjamini and Hochberg’s method for a strong control of the false discovery rate. Raw and preprocessed microarray data have been deposited at the Gene Expression Omnibus (accession number GSE37172, GSE39071).

## Results

### Expression of EpCAM in normal breast tissue and primary human mammary epithelial cells

**Figure 1:**
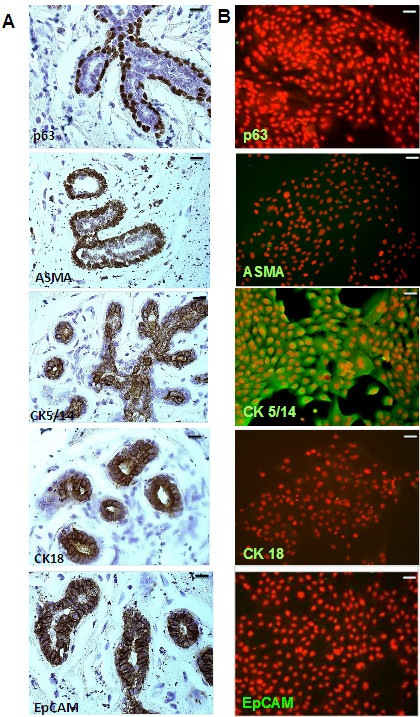
**Characterization of primary human mammary epithelial cells (HMECs).** Paraffin sections (**A**) or HMECs cultivated on collagen-coated cover slides (**B**) were stained with antibodies (markers) specific for myoepithelial (ASMA), basal/stem cells (p63), basal cells (CK5/14), and luminal cells (CK18). Immunohistochemical stainings revealed that *in vivo* EpCAM was expressed in basal and luminal cells of the breast, but not in myoepithelial cells. Immunofluorescence analysis revealed that HMECs stain for basal marker CK5/14, but were consistent negative for EpCAM, p63 and ASMA (n = 3). Magnification 200 ×; bars indicate 50 μm.

In the mammary gland all epithelial cells express EpCAM with the exception of myoepithelial cells. There were no significant changes in immunoreactivity between luminal and basal cells (Figure 1A). In clear contrast to all tumor samples analyzed, normal polarized epithelia had a strict localization of EpCAM on the basolateral membrane. This basolateral expression got lost in tumor cells of primary breast carcinoma and metastasis which showed clearly localization on the entire cell surface (Additional file 1: Figure S1). Primary epithelial cells from healthy breast tissue (HMECs) were analyzed for their phenotype by a panel of markers specific for myoepithelial (ASMA), progenitor (p63), basal (CK5/14) and luminal (CK18) epithelial cells. As expected, all HMECs lacked luminal (CK18-) or myoepithelial (ASMA-) markers, but displayed more a basal phenotype (p63-, CK5/14+). Interestingly, *in vitro* cultivated HMECs were negative for EpCAM in the immunofluorescence analysis (Figure 1B), although low transcript levels could be detected by qPCR analysis (data not shown).

### Adenoviral overexpression of EpCAM inhibited cell proliferation and migration in HMECs

**Figure 2:**
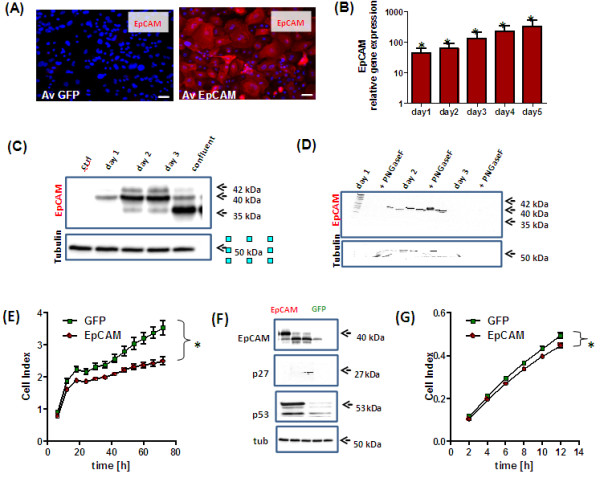
**Adenoviral overexpression of EpCAM in HMECs inhibits cell proliferation and migration.** HMECs (n = 3) were adenovirally transfected to overexpress EpCAM and GFP or GFP alone. A multiplicity of infection of 100 viruses/cell was used for all experiments. Overexpression of EpCAM was confirmed 48 h after transfection by Immunofluorescence (**A**). EpCAM was expressed on cell surface and cytoplasm (Phycoerythrin, red signal). Nuclei were counterstained with DAPI (blue signal, magnification 400×, bars indicate 25 μm). EpCAM overexpression was analyzed by real time PCR using GAPDH as housekeeping gene for normalization and GFP transfected cells as controls (**B**). As expected, overexpression resulted in a more than hundred-fold induction of EpCAM gene expression even 5 days after transfection. Protein expression was confirmed by Western Blot analysis (**C**). In comparison to control cells EpCAM was overexpressed as glycosylated isoform in proliferating cells and primarily as not glycosylated isoform in growth arrested HMECs. EpCAM glycosylation has been analyzed by enzymatic deglycosylation experiments with PNGaseF and subsequent Western Blot analysis. In all samples we observed a reduction of the 40-42 kDa glycosylated isoforms to the 35 kDa not glycosylated EpCAM isoform (**D**). Cell proliferation was analyzed in real time by the use the xCelligence system. EpCAM overexpression significantly inhibited cell proliferation (**E**). Western Blot analysis of cell cycle inhibition 48 h after EpCAM overexpression; p53 and p27^KIP1^ proteins were upregulated in EpCAM overexpressing cells. (**F**). Cell migration was monitored by xCelligence CIM plate system after adenoviral transfection of EpCAM or GFP (**G**). EpCAM overexpression also inhibited cell migration; stars indicate p values < 0.05.

Based on our observations that HMECs display low endogenous EpCAM expression in 2-dimensional cultures, we overexpressed the putative EpCAM oncogene and analyzed effects on cell proliferation and migration *in vitro*. Using a multiplicity of infection (MOI) of 100 viruses/cell we obtained a strong EpCAM expression in HMECs (Figure 2A) without any effects on cell viability (Additional file 2: Figure S2B). Noteworthy, next to the native EpCAM protein on plasma membrane (Additional file 2: Figure S2A) we found a lot of immunoreactive EpCAM in cytoplasmic organelles in our immunofluorescence analysis (Additional file 1: Figure S1D). These high amounts of cytoplasmic EpCAM might originate by overload of the intracellular vesicular traffic system with EpCAM or by a preferential detection of cytoplasmic EpCAM isoforms in our immunofuorescence analysis.

A transient, about hundred-fold overexpression was obtained over the observed time period of 5 days in all HMEC cultures (Figure 2B). EpCAM overexpression in HMECs was also confirmed on protein level by Western Blot analysis (Figure 2C). Interestingly, proliferating HMECs produced predominantly glycosylated isoforms (days 1 to 3), whereas in confluent and contact-inhibited cultures most of EpCAM protein was not glycosylated (Figure 2C). The presence of different EpCAM isoforms in HMECs was confirmed by enzymatic deglycosylation experiments with the enzyme PNGaseF and subsequent Western Blot analysis (Figure 2D). Under optimal mitotic stimulation EpCAM overexpression inhibited cell growth in proliferating HMECs as determined by the Real Time Cell Proliferation System (Figure 2E). In comparison to control cells, EpCAM transfected cells showed elevated expression of the tumor suppressor genes, p27^Kip1^ and p53 (Figure 2F). However, these changes were visible only as a post-transcriptional regulation, on the protein level. Gene expression levels of *TP53* and *p27*^*Kip1*^ did not significantly change after adenoviral transfection (Additionl file 2: Figure S2C). EpCAM overexpression resulted also in a slight, but significant inhibition of cell migration as observed by the real time cell migration measurement (Figure 2G).

### EpCAM expression is not induced by polarization processes in HMECs

**Figure 3:**
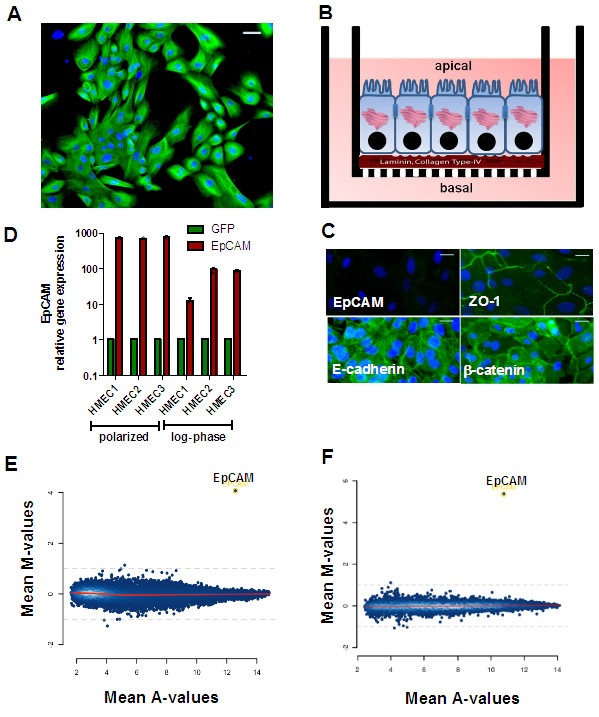
**Analysis of EpCAM target genes in HMECs.** HMECs were cultivated as mitotic subconfluent cultures (**A**) or for 10 days on Matrigel-coated transwells to induce polarization of epithelial monolayers (**B**). Then, cells were adenovirally transfected (MOI = 100) to overexpress GFP alone or EpCAM/GFP. Polarized HMECs cells did not express immunoreactive EpCAM protein, but the gap junction protein ZO-1, E-cadherin and β-catenin as determined by Immunofluorescence analysis (**C**, bars indicate 25 μm). Polarized cells and mitotic cultures (log-phase) of three donors (HMEC 1–3) were adenovirally transfected and EpCAM gene expression quantified 24 h later by real time PCR using GAPDH as internal house-keeping gene (**D**). MA-plot of genes regulated by EpCAM as determined by Affymetrix Gene analysis in mitotic standard cultures (**E**) or confluent polarized cultures of HMECs (**F**). Besides EpCAM no additional genes were significantly regulated in all three donors analyzed 20 h after transfection. Mean M-values indicate differential gene expression between EpCAM over-expressing and GFP transfected cells (log2 scale), Mean A-values indicate average expression of a gene in all microarrays. Stars indicate p values <0.05.

Although EpCAM expression was strictly basolateral in breast epithelia *in vivo*, it was not expressed in our *in vitro* cultures of HMECs (Figure 1B). Therefore, we concluded, that maintenance of cell polarity with functional tight- and gap- junctions is necessary for the expression of EpCAM and for further overexpression studies. HMECs were grown as mitotic cultures on collagen type I (Figure 3A) or as confluent, polarized monolayers on 0.4 μM transwell inserts coated with Matrigel (Figure 3B). Polarization of HMECs was controlled after 10 days by measurement of transepithelial resistance (860 ±50 Ω) and by immunofluorescence stainings for the tight junction marker ZO-1, and cell-cell contacts mediated by E-cadherin and membranous β-catenin. Cell-cell contact proteins E-cadherin and β-catenin, molecular interaction partners of EpCAM, were strongly expressed in polarized HMECs cultures (Figure 3C). However, we could not observe elevated EpCAM protein expression (Figure 3C).

### EpCAM overexpression does not alter gene expression profile of HMECs

HMECs grown as polarized cultures or under mitotic culture conditions were adenovirally transfected to overexpress EpCAM/GFP or GFP. As expected, transient transfection resulted in a strong overexpression of EpCAM in comparison to control cells (Figure 3D). Despite equal multiplicities of infection (MOI = 100) used for all transfections, EpCAM overexpression was stronger in polarized cells than in standard culture conditions. Based on our data on EpCAM protein expression (Figure 2C) we isolated mRNA 24 h after adenoviral transfection to identify genes directly regulated by EpCAM and not thereafter, by induction of the transcription factor p53. Apart from the clear overexpression of EpCAM, we did not observe any significant changes in the gene expression profile of HMECs under normal and polarized culture conditions (Figure 3E/F, Additional file 3: Table S1). These microarray data indicate that EpCAM overexpression alone does not directly affect gene transcription in HMECs either cultured in a polarized, tissue resembling culture model or under mitotic standard conditions.

### EpCAM antagonizes TGF-β1 induced growth arrest

**Figure 4:**
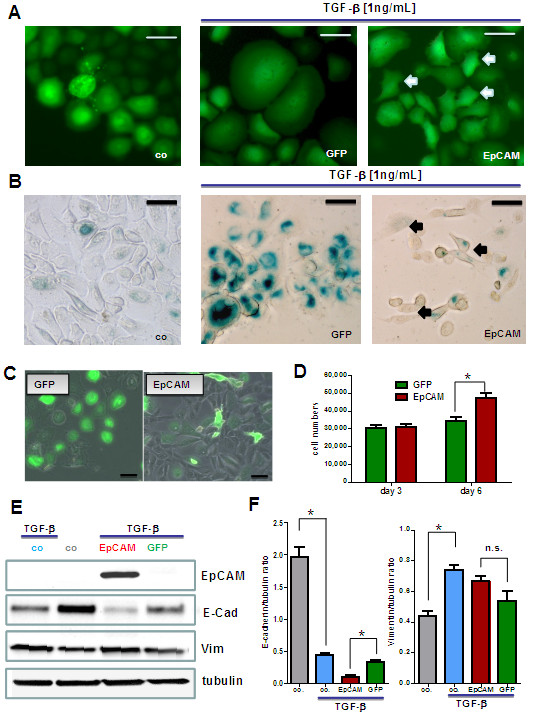
**EpCAM inhibits TGF-β1 induced terminal growth arrest and differentiation in HMECs.** Adenovirally transfected HMECs were stimulated with 1 ng/mL TGF-β1 to undergo terminal growth arrest and differentiation *in vitro*. (**A**) In comparison to control cells TGF-β1 treated GFP expressing cells got growth arrested, flat and enlarged. Populations of EpCAM transfected cells were protected from TGF-β1 and acquired a small cell body (white arrows). (**B**) In comparison to proliferating control cells TGF-β1 treated cells stained positive for senescence-associated beta galactosidase (SA-β-Gal, blue color), a marker for terminally arrested cells. EpCAM transfected cells were predominantly negative and acquired a more spindle shaped morphology (black arrows). (**C**) Long term cultures of transfected HMECs in the presence of TGF-β1. EpCAM transfected cells showed a higher proliferative capacity within the observed time window of 6 days than GFP controls. Bars indicate 25 μm. (**D**) Cell numbers were analyzed 1, 3 and 6 days after TGF-β stimulation by counting in a Buerker-Tuerk chamber. EpCAM transfected cells displayed significantly higher proliferative activities, *i.e.* higher cell counts after 6 days of growth. (**E**) Western Blot analysis of differentiation markers for epithelial mesenchymal transition (vimentin, E-cadherin). (**F**) EpCAM transfected HMECs show a downregulation of E-cadherin (E-cad) but no significant upregulation of vimentin (Vim) protein. Stars indicate p values <0.05.

TGF-β1 acts on epithelial cells as potent growth inhibitory factor and promotes differentiation processes. Basal cells stimulated with TGF-β1 stop proliferation within 3 days. In contrast to untreated control cells, displaying a small cell body and a strong light refracting morphology, TGF-β1 treated cells changed morphology and acquired an enlarged and flat cell body (Figure 4A/B, green signal). After EpCAM overexpression, TGF-β1 stimulated HMECs showed a higher percentage of cells with a small, strongly light-refracting morphology (Figure 4A, arrows). Moreover, HMECs treated with TGF-β1 underwent a terminal growth arrest and stained positively for senescence-associated beta galactosidase (SA-β-Gal; Figure 4B), a marker of cellular senescence. In clear contrast, upon simultaneously EpCAM overexpression, we could observe many cell clusters that were negative for SA-β-Gal (Figure 4B, arrows) indicating that cells were not growth arrested and maintained a longer capacity to proliferate.

### EpCAM down regulates E-cadherin and prolongs proliferative lifespan of HMECs

Long term cultures of HMECs in culture medium containing TGF-β1 were analyzed for differences between EpCAM/GFP and GFP overexpression. In the presence of the differentiation factor TGF-β1 EpCAM overexpressing cells were still able to proliferate and formed bigger cell clusters after 6 days *in vitro* (Figure 4C). GFP transfected control cells stopped cell divisions after 3 days and consisted predominantly of enlarged, flat and growth arrested cells (Figure 4C). Analysis of cell numbers revealed a significant increase in cell counts in EpCAM overexpressing cells 6 days after transfection (Figure 4D).

Additionally, we analyzed a panel of epithelial-to-mesenchymal transition (EMT) markers to define the phenotype of EpCAM overexpressing cells. In particular, HMECs showed down regulation of E-cadherin after TGF-β1 stimulation (Figure 4E, 4F). In clear contrast to GFP control cells, EpCAM-overexpressing cells showed an additional down regulation of E-cadherin (Figure 4E, 4F), which would explain the more spindle-shape phenotype of the cells (Figure 4A). Another marker of EMT, vimentin expression, did not increase significantly after EpCAM overexpression in direct comparison to GFP transfected control cells (Figure 4E, 4F).

### EpCAM overexpressing HMECs form bigger xenografts consisting of p63^high^ progenitor cells and lack luminal structure formation

**Figure 5:**
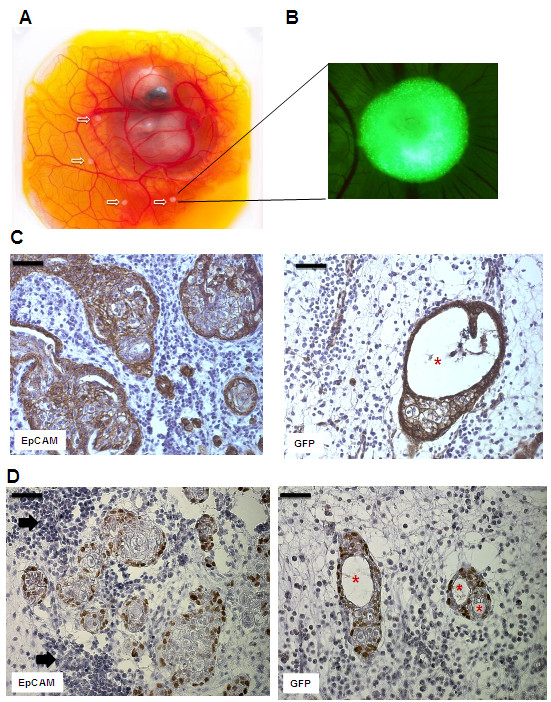
**EpCAM overexpression *in vivo *leads to hyperplastic cell growth without ductal lumen formation.** HMECs were transplanted together with matrigel into the chorioallantoic membrane (CAM) of chicken embryos and cell growth and morphology analyzed after 6 days. (**A**) Chicken embryos with HMEC xenografts. White arrows indicate transplants of HMECs in matrigel plugs (**B**) Fluorescence stereo microscope picture of a HMEC graft 6 days after *in vivo* growth. The green clusters (adenoviral GFP) indicate human cell-cell aggregates that growth inside the CAM (magnification 20×) (**C**) Immunohistochemical analysis of cross-sections of HMEC grafts in the CAM. Sections were stained with an antibody specific for human cadherin, thus detecting only human cells. In contrast to GFP transfected controls, EpCAM overexpressing grafts show bigger glandular structures that lack formation of lumen. (**D**) Immunohistochemical analysis of p63^high^ progenitor cells. Noteworthy, HMECs at the glandular base express the progenitor marker p63. EpCAM overexpressing clusters are surrounded by significant bigger clusters of chicken leukocytes (black arrows). Bars indicate 50 μm, asterisks indicate lumen.

Based on *in vitro* findings we analyzed effects of EpCAM overexpression in our *in vivo* model. Therefore, we transplanted HMEC xenografts onto chicken embryos to analyze *in vivo* growth (Figure 5A). Chicken embryos have only innate immune responses and thus, tolerate growth of human cells. Transfected HMECs were transplanted as growth factor-reduced matrigel drops containing TGF-β1. After 6 days *in vivo* growth, xenografts became well vascularized and human onplants could be visualized by expression of GFP (Figure 5B). Macroscopically, there were no significant changes in the size of HMEC onplants, however immunohistochemical analysis of invading cell clusters in the chicken chorioallantoic membrane tissue revealed morphological and quantitative differences. Cell clusters of GFP controls were smaller, less frequent and displayed significantly more lumen formation (Figure 5C, 5D, marked with asterisks). In contrast, EpCAM overexpressing HMECs formed bigger structures, with more frequent disseminating cell clusters and therefore, almost no lumen formation (Figure 5C). Higher cell numbers of EpCAM overexpressing HMEC grafts were also correlating with more p63^high^ progenitor cells/high power field (Figure 5D).

### EpCAM overexpression enhances cell proliferation in immortalized MCF-10A cells

**Figure 6:**
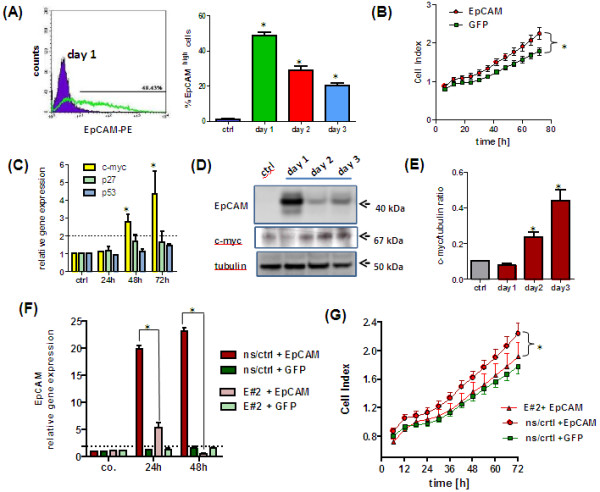
**EpCAM overexpression leads to upregulation of c-myc and increased cell proliferation in immortalized MCF10A human breast epithelial cells.** (**A**) Flow cytometric analysis of EpCAM expression in adenovirally transfected MCF10A cells. In comparison to GFP transfected controls, only EpCAM transfected cells show the immunoreactive protein on the cell membrane. (**B**) Overexpression of EpCAM results in a significant increase in cell proliferation under serum-reduced conditions. (**C**) Real time PCR analysis of *TP53*, *p27kip1* and *c-myc* gene expression in EpCAM transfected cells. Overexpression of EpCAM upregulated *c-myc* gene expression. (**D**) Western Blot analysis of EpCAM overexpression and upregulation of c-myc protein levels. Tubulin alpha served as internal loading control. (**E**) Densitometric analysis of c-myc to tubulin protein ratio. MCF10A cell lines were generated by a lentiviral system to have a stable expression of a non-silencing control (ns/crtl) or an EpCAM specific (E#2) shRNA. MCF10A ^ns/crtl^ and MCF10A ^E#2^ cells were adenovirally transfected to overexpress GFP or EpCAM/GFP. (**F**) In comparison to MCF10A ^ns/crtl^ cells MCF10A ^E#2^ cells were significantly downregulating EpCAM transcript levels 24 and 48 h after adenoviral transfection. (**G**) Real time cell proliferation of MCF10A ^E#2^ cells was significantly lower than those of MCF10A ^ns/crtl^ after adenoviral EpCAM overexpression. Stars indicate p values <0.05.

Based on our observation that EpCAM overexpression alone is not enough to reveal its oncogenic features and that tumorigenesis is a multistep process, we decided to use the immortalized breast epithelial cell line MCF-10A (p16^INK4a-/-)^ for additional investigations. MCF10A cells can be efficiently transduced with adenovirus to overexpress EpCAM, but loose EpCAM expression faster than HMECs (Figure 6A). In comparison to HMECs (Figure 2E), MCF10A with EpCAM overexpression show an increased cell proliferation (Figure 6B) and upregulation of *c-myc* gene expression (Figure 6C). Changes of c-myc expression could also be monitored on protein level (Figure 6D/E).

Moreover, MCF10A cell lines were generated by a lentiviral system to have a stable expression of a non-silencing control (ns/crtl) or an EpCAM specific (E#2) shRNA. Both cell lines, MCF10A ^ns/crtl^ and MCF10A ^E#2^, were adenovirally transfected to overexpress GFP or EpCAM/GFP. In comparison to MCF10A ^ns/crtl^ cells MCF10A ^E#2^ cells were significantly downregulating EpCAM transcript levels 24 and 48 h after adenoviral transfection (Figure 6F). Real time cell proliferation of MCF10A ^E#2^ cells was significantly lower than those of MCF10A ^ns/crtl^ after adenoviral EpCAM overexpression (Figure 6G). These data clearly indicate that EpCAM overexpression can enhance proliferation and c-myc levels in immortalized human breast epithelial cells.

## Discussion

EpCAM is a widely described tumor-associated antigen, stem cell and cancer stem cell marker [4,22-24]. Cancer stem cells with a high EpCAM expression are considered to be more malignant and more prone to give metastasis than those with a low expression [24,25]. Although EpCAM overexpression in breast cancer is correlated with aggressive behavior and decreased overall survival of patients [13,25-27], functions and effects of EpCAM overexpression in normal mammary epithelial cells, *i.e.* healthy tissue have not been described so far.

In normal breast epithelia EpCAM has a strict basolateral expression. Among all epithelial cell-types only myoepithelial cells are EpCAM-negative [28]. Tumor cells loosing cell-cell contacts and invading host tissue are also loosing the strict basolateral distribution of EpCAM and show more cytoplasmic and membranous staining [5,27]. Whether this is mediated by loss of cell polarity or by generation of translocated EpCAM isoforms is still under investigation [5,11,29]. Recent studies showed that glycosylation of EpCAM might affect stability and function of the protein [30]. Noteworthy, healthy tissue displays mainly weak expression of basic, not glycosylated EpCAM protein, whereas in tumor tissue, as well as in breast cancer cell lines, EpCAM is glycosylated and/or hyperglycosylated [30]. Differences in glycosylation we could also observe between highly mitotic cultures and growth arrested monolayers of transfected human mammary epithelial cells (HMECs).

*In vitro* cultivated HMECs showed no EpCAM protein expression, although gene transcripts could be detected by qPCR in a low abundance. Presumably, these cells loose expression under artificial *in vitro* conditions and loss of normal tissue polarity, since *in vivo* both basal/progenitor as well as differentiated luminal cells are strongly positive for immunoreactive EpCAM. Moreover, cell-cell adhesions in our HMECs are primarily mediated by E-cadherin, which has been described to be a counter player of EpCAM [10,18,31]. Typically, HMEC cultures age under mitotic stress and induce p16^INK4A^ and/or p53 [32,33]. Aberrant expression of oncogenes has been shown to induce cellular senescence (oncogene-induced senescence) by activation of the p53, p16/Rb or p27^Kip1^ checkpoint. These check-points of the cellular senescence program protect cells from oncogenic signaling, prevent immortalization and acquisition of genomic instabilities [34,35] and are very often inactivated in cancer cells. In comparison to control cells, overexpression of EpCAM led to inhibition of proliferation and migration in HMECs. This represents a frequently observed reaction of normal cells to an oncogenic stimulus. However, in contrast to effects described for oncogenic *ras* or the catalytic subunit of the telomerase *(TERT)* we did not observe a complete growth arrest mediated by induction of p16^INK4A^[35,36]. EpCAM transfected HMECs are inhibited in cell proliferation, but do not undergo a terminal growth arrest. This might be due to simultaneous upregulation and accumulation of p53 and the cell cycle inhibitor p27^Kip1^. A crosstalk between EpCAM and p53 has already been reported [37]. EpCAM gene expression is downregulated by p53 and loss of p53 leads to increased EpCAM expression and a more invasive phenotype in tumor cells [36,37].

EpCAM did not affect p53 or p27^Kip1^ gene transcription, upregulations were only visible on the protein level. Thus, EpCAM might induce changes in p53 protein by affecting posttranscriptional modifications processes or protein stability [38]. Moreover, p27^Kip1^ has been shown to inhibit Rho-A driven cell migration processes [39]. Thus, our HMECs upregulating p27^Kip1^ after EpCAM overexpression probably showed an inhibition of cell migration despite down regulation of the cell-cell adhesion molecule E-cadherin.

Against our expectations, EpCAM expression alone did not directly affect transcription of other genes in our HMEC culture models, although a signaling pathway, directly activated by EpCAM cleavage, has been previously described in pharyngeal cancer cells [35,36]. In fact, in HEK293 and FaDu tumor cell lines EpCAM has been reported to act directly on transcription of *c-myc* and *cyclins*[40,41]. We transfected growth arrested and polarized, as well as proliferating HMEC cultures and performed transcriptome analysis 24 h after overexpression of EpCAM. With this experimental approach we wanted to identify early genes directly regulated by EpCAM, before induction of the transcription factor p53 and its downstream genes. Both attempts gave no evidence that EpCAM overexpression is directly affecting gene expression profile of HMECs. Our data indicate that at least in primary HMECs overexpression of EpCAM, with absence of other oncogenes or mutations, has no immediate and direct effect on gene transcription. In fact, MCF10A, immortalized human epithelial cells having inactivation of the *INK4A* (p16) gene locus, respond to EpCAM overexpression by upregulating *c-myc* gene expression. Thus, we assume that other transforming stimuli have to act together with EpCAM to induce changes on gene transcription level.

In fact, EpCAM is primarily acting on cell-cell adhesion proteins such as E-cadherin, claudins, tetraspanins and CD44 [42]. Changes on their protein levels, localization on the cell membrane and interactions, might affect intracellular signaling pathways and kinase activities. Indeed, it has been recently reported that EpCAM affects protein kinase C signaling and cell migration processes during gastrulation in xenopus embryos [43].

HMECs are very sensitive to the cytokine TGF-β1 treatment [36]. This cytokine is able to inhibit cell proliferation and induce EMT differentiation processes in healthy epithelial cells [44,45]. When HMECs are transfected to overexpress EpCAM many clones acquire resistance to TGF-β1 induced growth arrest and display more spindle-shape phenotype. The underlying mechanism for increased resistance to TGF-β1 mediated growth arrest still remains to be investigated. Further, our *in vivo* studies support the concept of EpCAM overexpression as supportive factor for hyperplastic growth. EpCAM overexpression together with TFG-β1 and presumably other mitogenic factors present in Matrigel support hyperplastic growth and counteract growth arrest and terminal differentiation processes *in vivo*. We assume that HMECs with EpCAM overexpression gain longer proliferative capacities and acquire more resistances to growth inhibition due to activation of Wnt signaling. This increased stem cell signaling is supported by the observation that EpCAM overexpressing xenografts display an increased number of p63^+^ undifferentiated progenitor cells. This is of particular interest, since higher amounts of undifferentiated cells in mammary gland contribute to increased risk to develop breast cancer [46].

Moreover, EpCAM overexpression leads to stronger innate immune responses *in vivo*. EpCAM overexpressing xenografts attracts more neutrophils from host tissue, which would suggest that EpCAM is supporting migration processes of immune cells as described previously for dentritic cells [47]. However, further investigations are necessary to study effects of EpCAM expression on cancer cells in context of tumor immunology and microenvironment.

Thus, EpCAM overexpression might promote progression and metastasis of primary tumors. However, further studies are still needed to identify the underlying molecular mechanisms responsible for EpCAM overexpression in the context of TGF-β/Wnt signaling and breast cancer development. This background will allow us to understand the impact of EpCAM overexpression on transformation of breast epithelial cells and growth of breast cancer cells.

## Conclusions

EpCAM revealed oncogenic features in normal human breast cells, inducing resistance to TGF-β1-mediated growth arrest and supporting a cell phenotype with longer proliferative capacities *in vitro*. EpCAM overexpression resulted in hyperplastic growth and enhanced innate immune responses *in vivo*. Thus, we suggest that EpCAM acts as a prosurvival factor counteracting terminal differentiation processes in normal mammary glands.

### Ethical standards

This study on commercially available tissue sections and primary cells and cell lines did not need approval of the local ethic committee of the Medical University of Innsbruck. Handling of animals was conducted in compliance with Austrian State laws. Since the CAM assay, *i.e.* chicken embryo is an alternative model to replace animal experiments according to Austrian law an ethical approval is not required.

## Abbreviations

EpCAM: Epithelial cell adhesion molecule; TGF-β1: Transforming growth factor beta 1; CAM: Chorioallantoic membrane; HMECs: Human mammary epithelial cells; GFP: Green fluorescent protein; RT: Room temperature; RT-PCR: Reverse transcriptase-polymerase chain reaction; SA-β-gal: Senescence associated-beta galactosidase; MOI: Multiplicity of infection; EMT: Epithelial to mesenchymal transition.

## Competing interest

The authors declare that they have no competing interests.

## Authors’ contributions

AM carried out *in vitro* HMECs experiments, animal experiments and contributed substantially in the experimental design. GU designed all experiments and drafted the manuscript. JR and JL performed Affymetrix transcriptome analysis, GS and GG participated in study design and data interpretation. All authors read and approved the final manuscript.

## Supplementary Material

Additional file 1: Figure S1 Representative immunohistochemical staining of EpCAM in healthy tissue (**A**), primary invasive ductal carcinoma (**B**) and corresponding lymph node metastasis (**C**) Note: The strict basolateral expression of EpCAM in healthy glandular tissue gets lost in tumor cells in favor to a signal on the entire cell membrane. (**D**) Immunofluorescence analysis of EpCAM transfected HMECs. Adenoviral transfected cells were fixed, permeabilized and stained with an EpCAM specific antibody (green signal); nuclei were counter-stained with ToPRO-3 (red signal). Bars indicate 100 μm. Click here for file

Additional file 2: Figure S2 Flow cytometry analysis of EpCAM expression on cell membranes of viable HMECs after adenoviral transfection with EpCAM or GFP. EpCAM ^high^ cells were quantified in direct comparison to GFP transfected controls (**A**). Apoptosis/necrosis of HMECs was analyzed 48 h after transfection by staining of Annexin V/propidium iodide and flow cytometric analysis (**B**). Relative gene expression levels of *TP53, p27*^*Kip1*^ and *c-myc* were analyzed by RT-qPCR 24 to 72 h after adenoviral transfection and quantified in direct comparison to GFP transfected control cells (**C**). Stars indicate p values <0.05. Click here for file

Additional file 3: Table S1 Affymetrix chip analysis of HMECs (n = 3) adenovirally transfected to overexpress EpCAM. Gene expression in EpCAM transfected cells was quantified relative to respective control transfections with GFP. Mean ± standard deviation (SD). Click here for file
