# Supplementary figures and images for: EpCAM overexpression prolongs proliferative capacity of primary human breast epithelial cells and supports hyperplastic growth
Source: Mol Cancer. 2013 Jun 10;12:56. doi: 10.1186/1476-4598-12-56 (PMC3702434; doi:10.1186/1476-4598-12-56)

## Slide 1
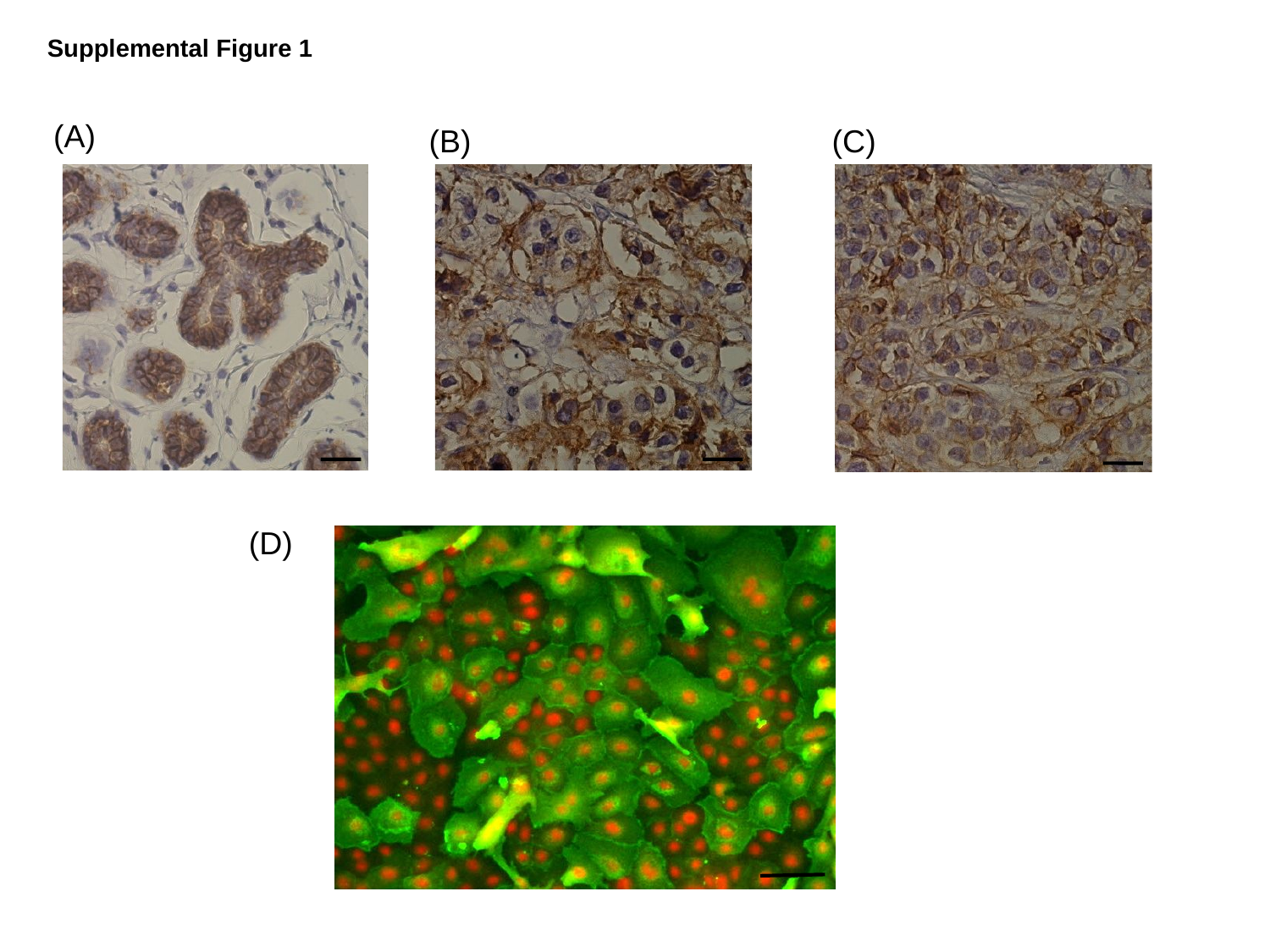

Supplemental Figure 1
(A)
(B)
(C)
(D)

Supplement: Additional file 1: Figure S1 — Representative immunohistochemical staining of EpCAM in healthy tissue (A), primary invasive ductal carcinoma (B) and corresponding lymph node metastasis (C) Note: The strict basolateral expression of EpCAM in healthy glandular tissue gets lost in tumor cells in favor to a signal on the entire cell membrane. (D) Immunofluorescence analysis of EpCAM transfected HMECs. Adenoviral transfected cells were fixed, permeabilized and stained with an EpCAM specific antibody (green signal); nuclei were counter-stained with ToPRO-3 (red signal). Bars indicate 100 μm. [file 1476-4598-12-56-S1.ppt]
