# Supplementary material for: EpCAM overexpression prolongs proliferative capacity of primary human breast epithelial cells and supports hyperplastic growth
Source: Mol Cancer. 2013 Jun 10;12:56. doi: 10.1186/1476-4598-12-56 (PMC3702434; doi:10.1186/1476-4598-12-56)
Supplement: Additional file 2: Figure S2 — Flow cytometry analysis of EpCAM expression on cell membranes of viable HMECs after adenoviral transfection with EpCAM or GFP. EpCAM high cells were quantified in direct comparison to GFP transfected controls (A). Apoptosis/necrosis of HMECs was analyzed 48 h after transfection by staining of Annexin V/propidium iodide and flow cytometric analysis (B). Relative gene expression levels of TP53, p27Kip1 and c-myc were analyzed by RT-qPCR 24 to 72 h after adenoviral transfection and quantified in direct comparison to GFP transfected control cells (C). Stars indicate p values <0.05. [file 1476-4598-12-56-S2.pptx]

## Slide 1
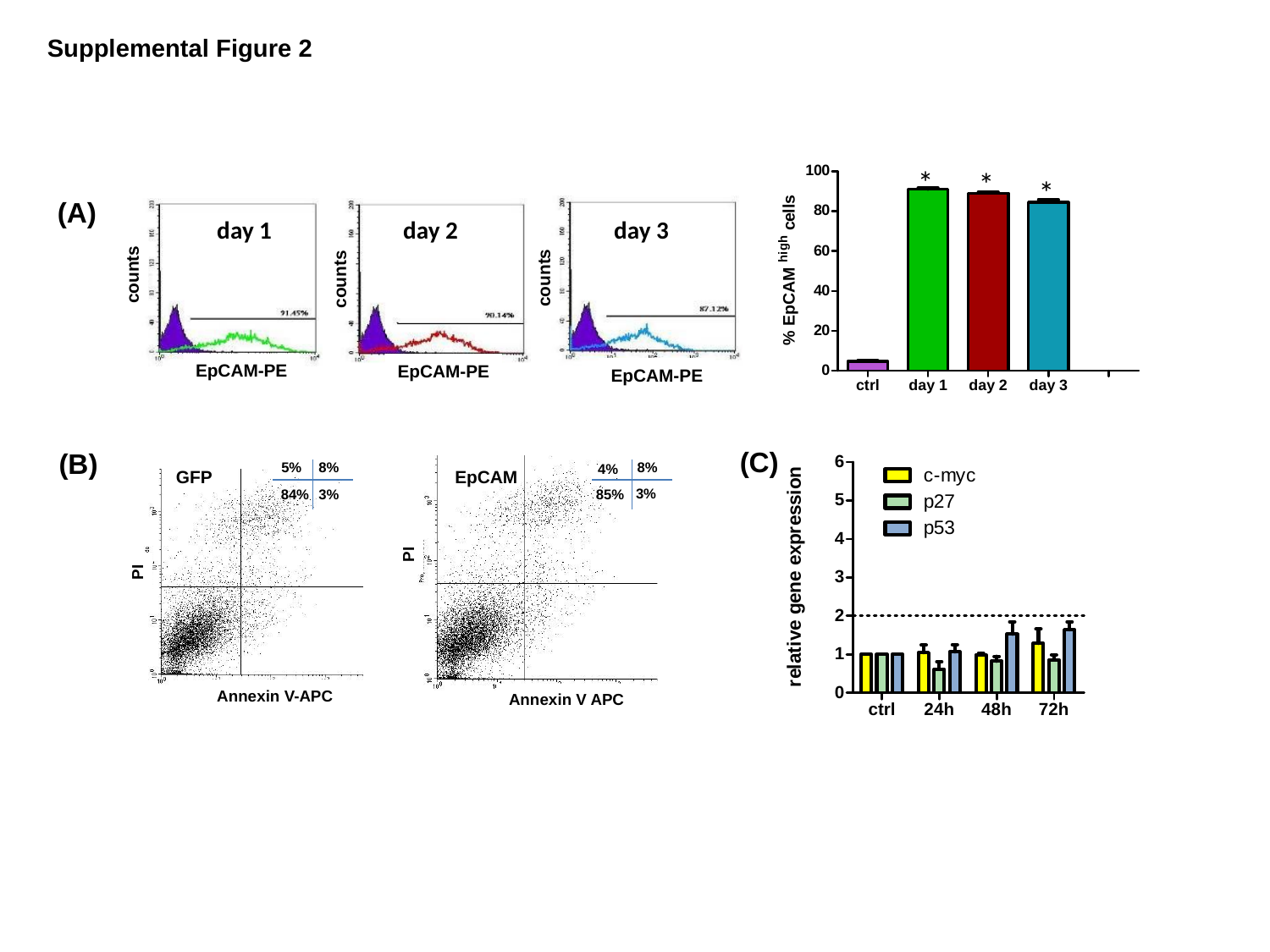

Supplemental Figure 2
*
*
*
(A)
day 1
day 2
day 3
counts
counts
counts
EpCAM-PE
EpCAM-PE
EpCAM-PE
(C)
(B)
5%
8%
8%
4%
GFP
EpCAM
3%
84%
3%
85%
PI
PI
Annexin V-APC
Annexin V APC
