# Supplementary material for: EpCAM overexpression prolongs proliferative capacity of primary human breast epithelial cells and supports hyperplastic growth
Source: Mol Cancer. 2013 Jun 10;12:56. doi: 10.1186/1476-4598-12-56 (PMC3702434; doi:10.1186/1476-4598-12-56)
Supplement: Additional file 3: Table S1 — Affymetrix chip analysis of HMECs (n = 3) adenovirally transfected to overexpress EpCAM. Gene expression in EpCAM transfected cells was quantified relative to respective control transfections with GFP. Mean ± standard deviation (SD). [file 1476-4598-12-56-S3.docx]

**Supplementary table 1**

Affymetrix chip analysis of HMECs (n=3) adenovirally transfected to overexpress EpCAM. Gene expression in EpCAM transfected cells was quantified relative to respective control transfections with GFP. Mean ± standard deviation (SD).

| Entrez | Symbol | Function | HMEC2 | HMEC3 | HMEC4 | Mean | SD |
| --- | --- | --- | --- | --- | --- | --- | --- |
|  |  |  |  |  |  |  |  |
|  |  | Signalling |  |  |  |  |  |
| 139728 | PNCK | CAM kinase | 0,55 | 0,54 | 0,64 | 0,58 | 0,06 |
| 4534 | MTM1 | dual-phosphatase | 0,46 | 0,59 | 0,59 | 0,54 | 0,08 |
| 5140 | PDE3B | phosphodiesterase | 0,70 | 0,70 | 0,70 | 0,70 | 0,00 |
| 1352 | COX10 | oxidase | 1,32 | 1,38 | 1,52 | 1,40 | 0,10 |
| 135112 | NCOA7 | Nuclear receptor | 0,62 | 0,85 | 0,72 | 0,72 | 0,12 |
|  |  |  |  |  |  |  |  |
|  |  | Growth factors |  |  |  |  |  |
| 7477 | WNT7B | growth factor | 0,61 | 0,74 | 0,84 | 0,72 | 0,11 |
| 7471 | WNT1 | growth factor | 0,86 | 0,94 | 0,94 | 0,92 | 0,04 |
| 7424 | VEGFC | growth factor | 0,92 | 0,85 | 0,91 | 0,89 | 0,04 |
| 1950 | EGF | growth factor | 0,81 | 1,09 | 1,00 | 0,96 | 0,14 |
| 23753 | SDF2L1 | growth factor | 1,15 | 1,37 | 1,51 | 1,33 | 0,18 |
|  |  |  |  |  |  |  |  |
|  |  | Proliferation |  |  |  |  |  |
| 4609 | MYC | transcription factor | 0,88 | 0,96 | 0,98 | 0,94 | 0,06 |
| 3725 | JUN | transcription factor | 1,02 | 0,91 | 1,07 | 1,00 | 0,08 |
| 1018 | CDK3 | protein kinase | 0,84 | 0,92 | 0,87 | 0,88 | 0,04 |
| 896 | CCND3 | cyclin | 0,87 | 0,83 | 0,91 | 0,87 | 0,04 |
| 85417 | CCNB3 | cyclin | 0,80 | 0,77 | 0,84 | 0,80 | 0,04 |
|  |  |  |  |  |  |  |  |
|  |  | Cytoskeleton and ECM |  |  |  |  |  |
| 4072 | EPCAM | adhesion molecule | 58,54 | 43,18 | 56,62 | 52,3 | 8,37 |
| 999 | E-cadherin | adhesion molecule | 1,10 | 0,98 | 0,98 | 1,02 | 0,07 |
| 5328 | PLAU | serin-protease | 0,82 | 0,84 | 0,83 | 0,83 | 0,01 |
| 83943 | IMMP2L | metalloprotease | 1,00 | 0,75 | 0,79 | 0,84 | 0,14 |
| 11174 | ADAMTS6 | metallopeptidase | 0,86 | 0,76 | 0,53 | 0,70 | 0,17 |
